# High fidelity computational simulation of thrombus formation in Thoratec HeartMate II continuous flow ventricular assist device

**DOI:** 10.1038/srep38025

**Published:** 2016-12-01

**Authors:** Wei-Tao Wu, Fang Yang, Jingchun Wu, Nadine Aubry, Mehrdad Massoudi, James F. Antaki

**Affiliations:** 1 Department of Biomedical Engineering, Carnegie Mellon University, Pittsburgh, PA, 15213, USA; 2 Key Laboratory for Molecular Enzymology and Engineering of Ministry of Education, Jilin University, Changchun 130012, China; 3 Advanced Design Optimization, Irvine, CA, 92618, USA; 4 Department of Mechanical Engineering, Northeastern University, Boston, MA, 02115, USA; 5 U. S. Department of Energy, National Energy Technology Laboratory (NETL), PA, 15236, USA

## Abstract

Continuous flow ventricular assist devices (cfVADs) provide a life-saving therapy for severe heart failure. However, in recent years, the incidence of device-related thrombosis (resulting in stroke, device-exchange surgery or premature death) has been increasing dramatically, which has alarmed both the medical community and the FDA. The objective of this study was to gain improved understanding of the initiation and progression of thrombosis in one of the most commonly used cfVADs, the Thoratec HeartMate II. A computational fluid dynamics simulation (CFD) was performed using our recently updated mathematical model of thrombosis. The patterns of deposition predicted by simulation agreed well with clinical observations. Furthermore, thrombus accumulation was found to increase with decreased flow rate, and can be completely suppressed by the application of anticoagulants and/or improvement of surface chemistry. To our knowledge, this is the first simulation to explicitly model the processes of platelet deposition and thrombus growth in a continuous flow blood pump and thereby replicate patterns of deposition observed clinically. The use of this simulation tool over a range of hemodynamic, hematological, and anticoagulation conditions could assist physicians to personalize clinical management to mitigate the risk of thrombosis. It may also contribute to the design of future VADs that are less thrombogenic.

Ventricular assist devices (VADs) offer a life-saving option for a great proportion of the 100,000 to 300,000 advanced heart failure patients in the United States^1^,^2^. However, this treatment is not exempt of serious adverse events. In particular, the current generation of these devices, so-called *continuous flow* VADs (cfVADs), have exhibited severe complications related to thrombosis, such as bleeding and stroke, that may negate the benefits of this therapy, and may require a subsequent pump-exchange surgery or lead to premature death^3^,^4^. In recent years, the rate of pump thrombosis has been increasing dramatically, which has raised grave concerns by the medical community^5^,^6^,^7^ and the FDA^8^. Although the inherent risk of thrombosis in these devices is well known, the precise mechanisms responsible for its initiation and progression have not yet been fully understood. Consequently, it is difficult to define best practices to ameliorate the risk of thrombosis without introducing other undesirable outcomes such as bleeding.

In general, it is likely that all three elements of Virchow’s triad play a role in pump thrombosis. But the complexities of the flow fields in these devices, combined with diverse biochemical, pharmacological, and biomaterial processes confound any attempt to seek a statistical model of thrombosis. Therefore, there is a need for a numerical predictive tool to supplant, or compliment, experimental trial-and-error. This has motivated our group over the past fifteen years to incrementally improve the fidelity of a multi-constituent model by adding important hemodynamic and biochemical pathways that are most relevant to blood-wetted devices. This paper reports the application of our most recent mathematical model to predict patterns of thrombus deposition that have been observed clinically in one of the most commonly used continuous flow VADs, the Thoratec HeartMate II (HMII).

## Methods

### Governing Equations

The thrombosis model presented here includes two groups of governing equations: equations of motion that determine the pressure and velocity fields and a set of coupled convection-diffusion-reaction (CDR) equations that govern the transport and inter-conversion of chemical and biological species – both within the thrombus and in the free stream. The mathematical details of this model have been recently reported in prior publications^9^,^10^, and are briefly summarized below.

#### Equations of Motion

Blood is treated as a multi-constituent mixture comprised of (1) a linear (Newtonian) fluid phase representing plasma and red blood cells (RBCs) and (2) a porous solid phase representing thrombus. The fluid phase, in the inertial frame of reference, is governed by the following continuity and momentum equations that also include interaction with the thrombus phase:


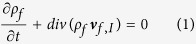



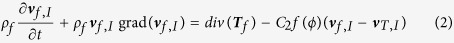



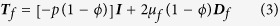


where *p* is the pressure, ***D***_*f*_ is the symmetric part of the velocity gradient, and *μ*_*f*_ consists of both the dynamic viscosity (=3.5cP) and the turbulent eddy viscosity^11^. A scalar field *ϕ* is introduced to represent the volume fraction of deposited platelets (thrombus) at any given point in space. The density of the fluid phase is defined in terms of the volume fraction according to:


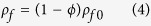


where *ρ*_*f*0_ is the density of the pure fluid phase (=1060 kg/m^3^), ***v***_*f,I*_ and ***v***_*T,I*_ are the velocity of the fluid and thrombus phases, respectively. The subscript *I* refers to the inertial frame of reference. The term *C*_2_*f*(*ϕ*)(***v***_*f*_ − *v*_*T*_) is the resistance force on the fluid phase imposed by the thrombus, where the coefficient *C*_2_ = 2 × 10^9^ *kg*/(*m*^3^*s*) is computed by assuming the deposited platelets behave like densely compact particles, as described by Johnson *et al*.^12^ and Wu *et al*.^13^. The function *f*(*ϕ*) = *ϕ*(1 + 6.5*ϕ*) is the hindrance function, which is obtained from the generalization of the interaction force from a single particle to an assemblage of particles^14^. Within the rotating impeller section of the pump, the above continuity and momentum equations can be expressed as follows:


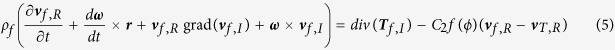



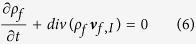


where ***v***_*f,R*_ = ***v***_*f,I*_ − ***ω*** × ***r*** is the velocity in rotating frame of reference, ***ω*** is the angular velocity of the impeller and ***r*** is the polar coordinate. For the thrombus phase, the only mechanical behavior we consider is the resistance force that it imparts on the fluid phase. Therefore, it does not require its own constitutive equation for stress. It should be noted that the velocity of the thrombus ***v***_*T*_ depends on the frame of reference to which it is attached. If attached to anywhere on the rotor, then ***v***_*T,I*_ = −***ω*** × ***r*** and if attached to a static wall ***v***_*T,I*_ = 0. Conversely*, **v***_*T,R*_ = 0, if attached to the rotor, and ***v***_*T,R*_ = −***ω*** × ***r*** if attached to a static wall.

### Chemical and biological species

**Figure 1:**
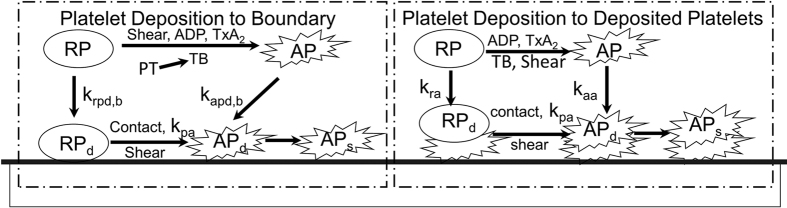
Schematic depiction of the thrombosis model, comprised of platelet deposition, aggregation, and stabilization. RP: resting platelet, AP: activated platelet, RP_d_ and AP_d_: *deposited* resting and active platelets, AP_s_: *stabilized* deposited active platelets. Agonists that cause activation (RP to AP) are adenosine diphosphate, ADP; thromboxane A2, TxA2; shear, and thrombin, TB – which is synthesized from prothrombin (PT). The suffix b refers to the reaction with the boundary (surface). The constants k_pa_, k_ra_, k_aa_, k_rpd,b_, k_apd,b_, refer to the reaction rates for inter-conversion of the associated platelet states.

Based on the continuum assumptions for blood, thrombosis is modeled using a system of convection-diffusion-reaction equations. As shown in Fig. 1, the model of thrombus formation accounts for the transport of ten (10) chemical and biological species, summarized in Table 1. These include five categories (states) of platelets (1) **RP**: resting platelets (in the flow field); (2) **AP**: activated platelets, which we assume have released their granules and become reactive; (3) **RP**_**d**_: deposited (trapped) resting platelets, (4) **AP**_**d**_: deposited active platelets, and (5) **AP**_**s**_: deposited and stabilized platelets. An additional five biochemical species include (1) **a**_**pr**_**(ADP)**: platelet-released agonists, (2) **a**_**ps**_**(TxA2)**: platelet-synthesized agonists, which can be inhibited via first-order reactions; (3) **PT:** prothrombin; (4) **T:** thrombin, synthesized from prothrombin on the platelet phospholipid membrane; and (5) **AT:** anti-thrombin III (ATIII), which inhibits thrombin and whose action is catalyzed by heparin via the kinetic model of Griffith^15^,^16^.

As shown in Fig. 1, the model attempts to simulate the following mechanisms occurring in blood. 1. *Platelet Activation*: RP can be converted to be AP through exposure to critical levels of biochemical agonists, namely adenosine diphosphate (ADP), thromboxane A2 (TxA2), thrombin (TB), and shear. It is assumed that this conversion causes the platelets to become adherent to biomaterial surfaces and to other platelets already deposited. 2. *Agonists generation*: The release of granules (ADP) is caused by the conversion of AP to RP; TxA2 is synthesized by AP; thrombin (TB) is synthesized from prothrombin (PT) on the platelet phospholipid membrane. 3. *Platelet Deposition*: Both RP and AP in the free stream are capable of depositing onto a surface, such as a channel wall, whereupon they are designated RP_d_ and AP_d_, respectively. This process is mathematically modeled by a reaction flux at the boundary. Therefore, the adhesion property of AP/RP to different biomaterials can be described by different levels of the reaction flux. 4. *Thrombus Propagation*: When a given volume in space is fully occupied by the thrombus, i.e. the volume fraction of the deposited platelets (RP_d_ and AP_d_) reaches a specified value, the subsequent deposition will propagate to an adjacent space. 5. *Thrombus Dissolution or Erosion*: The shear stress exerted by the fluid phase is capable of cleaning deposited platelets, thereby corresponding to the phenomenon known as surface “washing”. Mathematically, this was modeled by a thrombus clearance term that is related to the shear stress. 6. *Thrombus Inhibition*: Anti-thrombin III in the free stream and within the thrombus may bind to thrombin, thus neutralizing the effect of thrombin in activating additional platelets. Griffith’s template model is applied for the kinetics of thrombin inactivation by ATIII. The inhibition of ADP and TxA2 are modeled by first-order reaction rate constants. 7. *Thrombus Stabilization*: Newly deposited platelets are converted by a constant rate to form a stable clot which cannot be removed by the hydrodynamic force. 8. *Thrombus-fluid interaction*: As the thrombus accumulates in space, the flow field of blood will be altered due to the porous/solid properties of the thrombus. The permeability of the thrombus is inversely related to the volume fraction of the deposited platelets. As represented in Equation (2) above, this is computed as a resistance force to the fluid flow, analogous to the behavior of packed granular slurries. Additional details and descriptions of the models are provided in Wu *et al*.^9^,^10^.

With the above assumptions, the transport of the species in the flow field is described by ten convection-diffusion-reaction equations^15^,^16^ of the form:


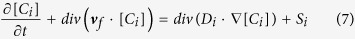


where [*C*_*i*_] is the concentration of species *i*, and *S*_*i*_ is a reaction source term for species *i*. *D*_*i*_ is the diffusivity of species *i*, given by:


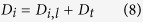


where *D*_*i,l*_ refers to the laminar (molecular) diffusivity and *D*_*t*_ is the turbulent (eddy) contributions. The latter is computed as


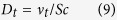


where *v*_*t*_ is the turbulent momentum diffusivity^11^, and *Sc* is the Schmidt number which is chosen as 0.9 in current study. Deposited platelets (RP_d_, AP_d_, and AP_s_) do not have convection and diffusion terms associated with them, and are each governed by a concentration rate equation:


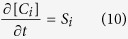


Platelet-surface adhesion and all the other reactions are modeled by surface-flux boundary conditions. Here, similar to the reaction terms in the internal domain, a negative and a positive flux corresponds to consumption and generation, respectively. Additional details of our thrombosis model are provided in a prior publication, which includes the physical description of all the terms as well as the values and origins of all coefficients^9^,^10^.

**Figure 2:**
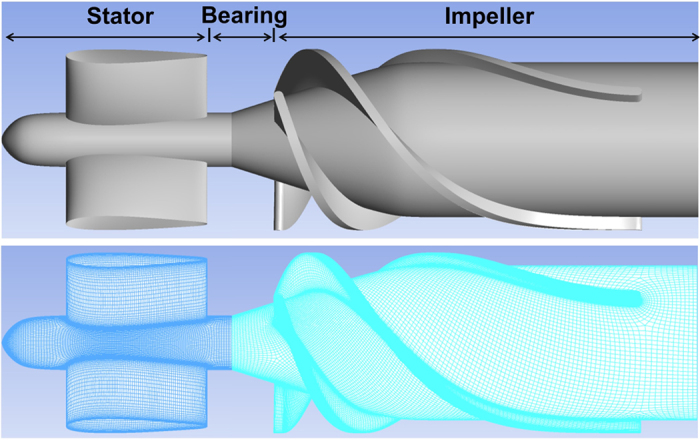
Computational domain encompassing the geometry(top) and mesh(bottom) of the upstream portion of the HeartMate II pump.

The above model was simulated on a high performance PC workstation (Dell T7910), using the libraries of OpenFOAM. The computational domain was comprised of two subdomains corresponding to the upstream flow straightener and the upstream portion of the impeller of the Thoratec HeartMate II. (See Fig. 2.) This was the primary region of interest for which clinical data was available. The remainder of the flow path was omitted to save computational cost. The mesh was generated using our internally developed automatic elliptic mesh generation system, ADO_Mesh (Advanced Design Optimization, Irvine, CA), with a total of 2.3 million elements. All simulations were solved with an unsteady (transient) solver and the *k* − *ω* model was applied to account for turbulence^11^,^17^. The rotational speed of the impeller was prescribed at the nominal rate of 9000 RPM. All other components including the shroud were considered static. The interface coupling between the rotational and stationary domains was modeled using the AMIcyclic boundary condition. At the inlet, a uniform velocity profile was applied and the outlet pressure boundary condition was set as 0 Pa. The corresponding boundary conditions for platelets and biochemical species are listed in Table 1. Additional details and descriptions of the physical meaning of each of these terms in Table 1 are provided in Wu *et al*.^9^,^10^.

Clinically, the process of thrombosis occurs over the course of hours to days. However, it would be computationally prohibitive to perform a simulation for a corresponding time scale (due to the large number of elements and small time step.) Therefore, to compare the simulation results (on the order of minutes) to clinical observations (on the order of hours) we scaled the reaction rates *k*_*ra*_, *k*_*aa*_, *k*_*rpdb*_ and *k*_*apdb*_ by a factor of 30. In the results and discussion sections, all the time of simulation results are also scaled by a factor of 30.

## Results

**Figure 3:**
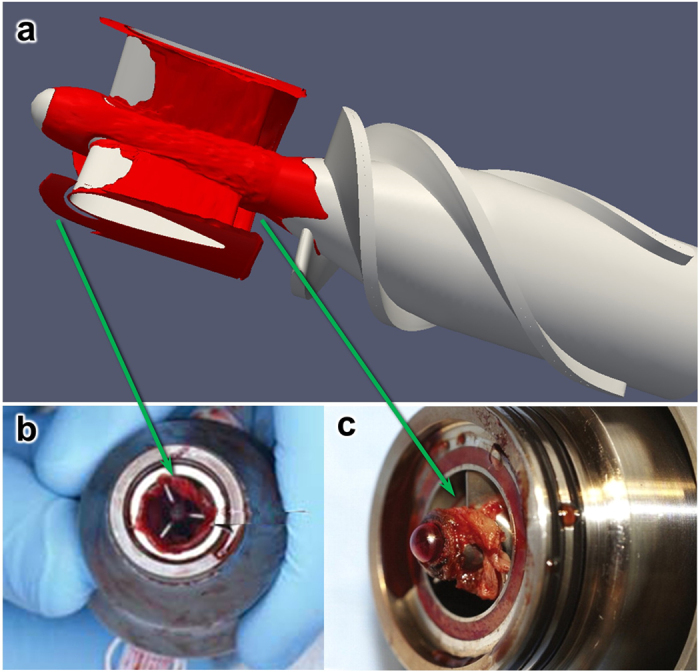
(**a**) Simulated thrombus deposition (in red) after 6.25 hours, corresponding to a flow rate of 4.5 L/min, and a rotational speed of 9000 RPM; (**b**) and (**c**): Clinical observation of thrombus deposition in HeartMate II removed from patients^18^,^19^. The correspondence is most remarkable upstream of the inlet straightener vane (**b**), and surrounding the front bearing (**c**). Figures (**b**) and (**c**) are used by permission.

**Figure 4:**
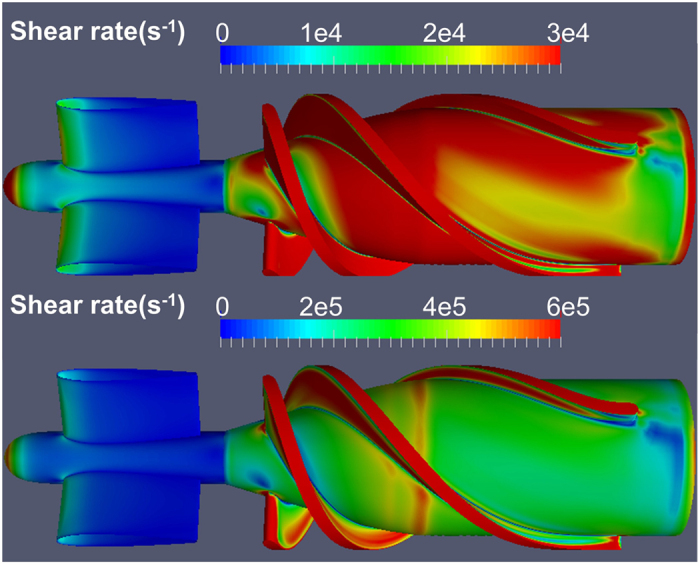
Shear rate on surfaces of the flow straightener and impeller (4.5 L/min, 9000 RPM). Color scales chosen to accentuate the hub surfaces (top) and impeller blades (bottom).

Figure 3 shows the simulated thrombus distribution in the upstream stator and impeller regions of the HeartMate II after 6.25 hours, at the flow rate of 4.5 L/min and rotational speed of 9000 RPM. This simulation required approximately 120 hours (5 days) using 40 CPUs (2.4 GHz). All the coefficients of the model were derived from previously reported values by Wu *et al*.^9^, with one exception, namely the characteristic platelet-platelet cleavage shear stresses *τ*_*sc*_ which was reduced by half (from 30 to 15 *dyne cm*^−2^), a reduction factor determined by optimization to better replicate the experimental results. The figure illustrates the regions where the thrombus preferentially accumulates, including the majority of the upstream flow straightener hub, the rear of the straightener vanes and the upstream bearing region. This finding is in good agreement with the regions in which the thrombus has been reported clinically^18^,^19^,^20^ (See Fig. 3b and c). Conversely, the simulation also predicted the regions in which the thrombus was *absent*, such as the leading edge of the blades of the inlet straightener vane. These correspond to regions of higher shear stress, which accentuates the erosion rate of deposited platelets (See Fig. 4).

**Figure 5:**
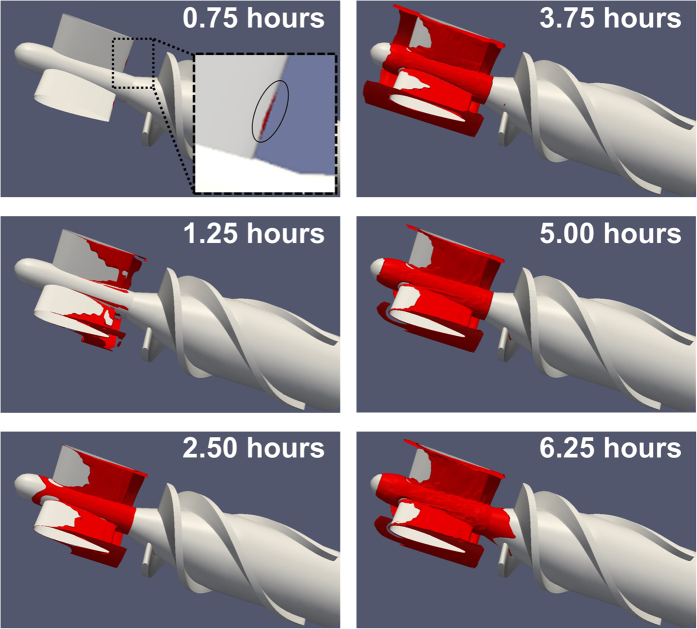
Progression of thrombus deposition in the upstream portion of the HeartMate II blood pump, corresponding to 0.75, 1.25, 2.50, 3.75, 5.00 and 6.25 hours in real-time. (For a flow rate of 4.5 L/min, and a rotational speed of impeller of 9000 RPM).

**Figure 6:**
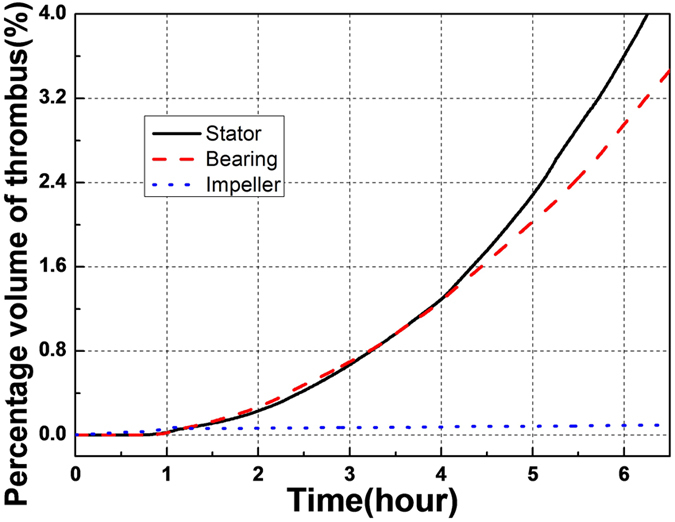
Thrombus accumulation in the three regions of the blood pump for a flow rate of 4.5 LPM and a rotational speed of 9000 RPM. Percentage volume of thrombus here refers to the proportion of the fluid subdomain occupied by thrombus.

Figure 5 shows the evolution of the thrombus over time, which indicates that the thrombus initiated at the rear of the inlet straightener vane and gradually propagated both downstream into the bearing region and upstream along the straightener blades. The initial thrombus deposition provides a substrate for attachment of incoming platelets and generates biochemical agonists that activate surrounding platelets. This positive feedback is also illustrated in Fig. 6 which provides a quantification of the total accumulation of deposited platelets in different regions of the pump as a function of time. It can be observed that deposition in the stator and bearing regions exhibits an interesting phenomenon that during the initial 1.25 hours, the thrombus accumulation is quite slow but accelerates after the thrombus accumulation reaches a critical level. Conversely, the deposition on the impeller surfaces never achieves a critical level of deposition (within the duration of our simulation, namely 6.25 hr).

### Effect of the flow rate on pump thrombosis

**Figure 7:**
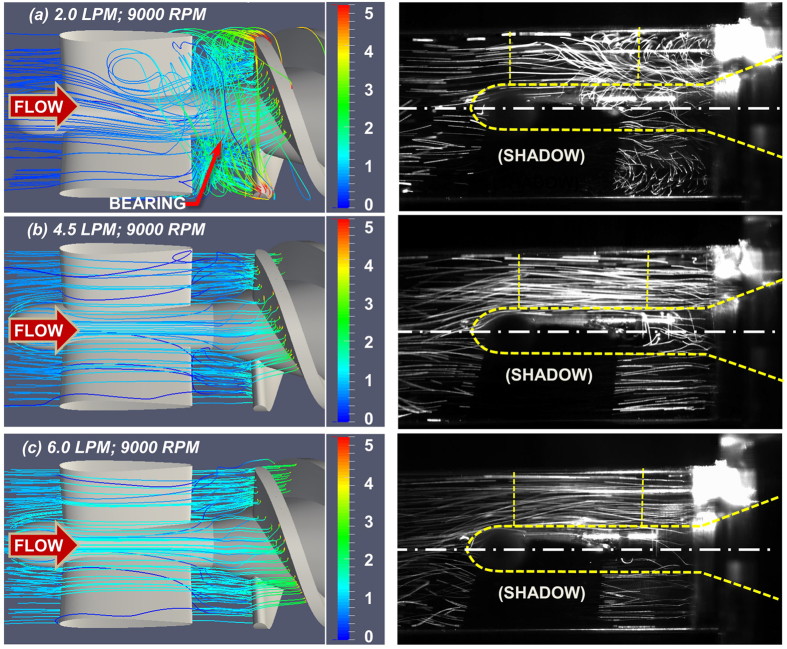
Numerical simulation (left) and fluorescent particle path lines (right) 21 **in the upstream region of the HeartMate II flow path.** The rotational speed is 9000 RPM in all three cases. Path lines are colored by velocity magnitude (m/s). (Experimental images published with permission).

To evaluate the influence of off-design conditions on thrombus deposition, simulations were performed for three different volumetric flow rates: the nominal flow rate (4.5 LPM), a lower flow rate (2.0 LPM), and a greater flow rate (6.0 LPM), while maintaining the rotational speed at 9000 RPM. The simulated velocity fields correspond to *in-vitro* experimental observations reported previously by flow visualization^21^ (See Fig. 7).

**Figure 8:**
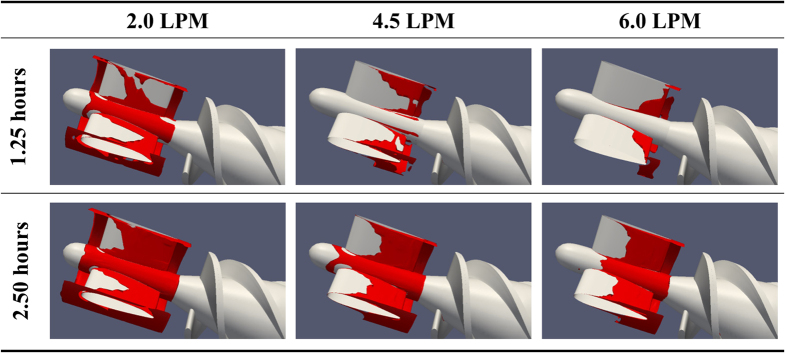
Thrombus deposition in the flow straightener and the impeller for three flow rates. For a rotational speed of 9000 RPM in all three cases.

**Figure 9:**
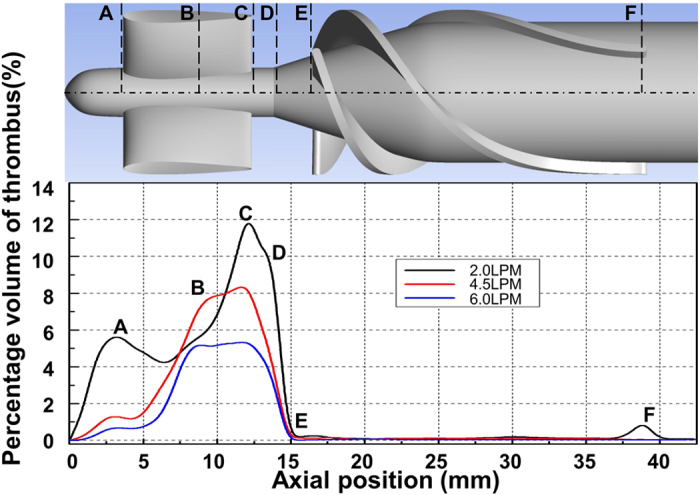
Thrombus accumulation (at t = 6.25 hours) along axial direction at different flow rates with 9000 RPM. The percentage volume of thrombus here is the proportion of the local fluid domain along axial direction occupied by thrombus. The axial position of the lines shown in the figure are: A = 3.8 mm, B = 8.0 mm, C = 12.9 mm, D = 14.2 mm, E = 16.5 mm and F = 39.0 mm.

Figure 8 shows the thrombus deposition in the straightener vane and bearing regions for the three volumetric flow rates studied. It can be observed that the rate of the thrombus deposition is inversely related to the flow rate. By referring to Fig. 9 and Table 2, which illustrate the axial distribution of the total thrombus deposition (at 6.25 h), it is clear that increasing the flow rate above the nominal flow rate (from 4.5 to 6.0 LPM) reduces the deposition in the stator and bearing regions uniformly. However, a reduction of the flow rate (from 4.5 to 2.0 LPM) both increases the overall deposition and significantly amplifies the deposition at the leading and trailing edges of the stator blade. Furthermore, this low flow rate causes deposition to initiate at the trailing edge of the impeller blade.

### Effect of heparin and material chemistry on pump thrombosis

**Figure 10:**
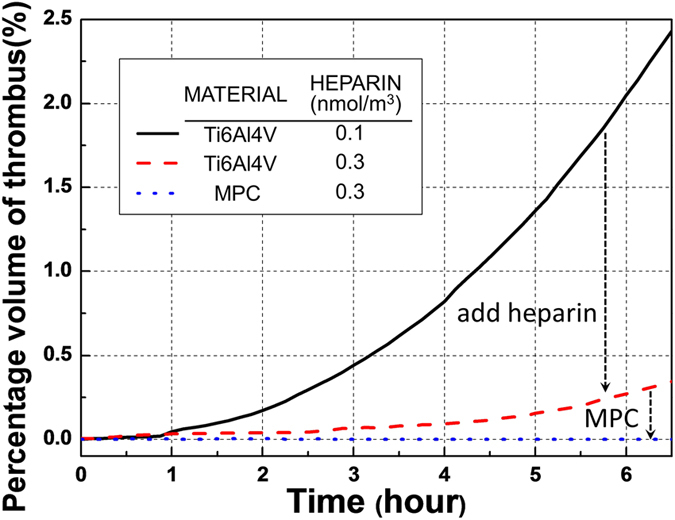
Effect of heparin and pump surface chemistry on thrombus accumulation. The percentage volume of thrombus here is the ratio of the entire fluid domain occupied by thrombus.

Additional simulations were performed to investigate the beneficial effects of altering the chemistry of the blood-wetted surfaces and the addition of heparin. Figure 10 provides simulation results of the total platelet deposition as a function of time for two hypothetical test cases corresponding to a bolus infusion of heparin, and substitution of the Ti6Al4V blood-contacting material with MPC (2-methacryloyloxyethyl phosphorylcholine). In the former case, the circulating heparin was increased from the baseline level 0.1 nmol/m^3^ to 0.3 nmol/m^3^ (corresponding approximately to a bolus infusion of 80 IU/kg). The latter case was simulated by reducing the platelet-surface deposition rate, *k*_*apdb*_, and platelet-surface cleavage (shear cleaning) constant, *τ*_*scb*_, by a factor of 10. Figure 10 illustrates a dramatic reduction of the deposition rate due to the administration of heparin, and virtually complete elimination of deposition due to combined effect of MPC coating and heparin.

## Discussion

The factors responsible for the recent escalation of thrombosis in the HeartMate II have evaded a definitive explanation^5^. This illustrates a persisting problem when developing any blood-wetted device. Despite best efforts to mitigate thrombosis during pre-clinical *in-vivo* validation and verification, new, unanticipated risk factors are revealed when introduced to humans. Unfortunately, there is no animal model that accurately reflects the milieu of pro-thrombotic factors that exists in patients with severe heart failure and multiple co-morbidities. Yet, in the clinical setting it is neither feasible, nor ethical, to investigate the myriad of independent device-related and patient-related variables that contribute to thrombosis. Hence is the motivation for a mathematical model: to enable *virtual* experimentation in-silico wherein hypotheses can be tested and treatments optimized rapidly, inexpensively, and without unwittingly injuring patients.

Over the past decades, a great deal of effort has been expended by many investigators to improve the performance of computational fluid dynamic models for continuous flow blood pumps^22^,^23^,^24^. Likewise great progress has been made in uncovering the mechanisms and feedback pathways for platelet activation and interaction with biomaterial surfaces^25^,^26^,^27^,^28^. Although some pathways (e.g. involving vonWillebrand factor) are not yet fully understood, there is now a great opportunity to combine the latest models of hemodynamics, platelet activation, transport, and anticoagulation into one high-fidelity model of thrombosis.

This is not meant to dismiss the value of less complicated approaches. For example, examination of flow patterns alone can reveal regions of flow recirculation and stagnation that are potentially thrombogenic. This can be accomplished both experimentally, through flow visualization, or numerically through computational fluid dynamics analysis. For instance, a recent CFD study by Chiu *et al*.^29^ performed a qualitative analysis of particle pathlines in the HeartMate II, and observed correlations between regions of recirculation zones and stagnant platelet trajectories with reported thrombus formation patterns. Yang *et al*.^21^ performed an analogous high-speed flow visualization of the upstream region of the HeartMate II under unsteady conditions, observing a toroidal vortex near the front bearing that corresponds to the thrombus deposition reported clinically. Both experimental and numerical analyses can elucidate an increased *risk* of thrombosis, but cannot provide definitive insight to the individual roles of hemodynamics, surface chemistry, and blood elements. Consequently, it is not uncommon for devices to be explanted from patients completely *free of thrombus* – despite the prevalence of pro-thrombotic flow patterns.

The interpretation of the thrombogenic potential of flow patterns is not trivial because they are highly three-dimensional, unsteady, and often chaotic. For example, it was somewhat counter-intuitive that the initial thrombus deposition in the present study occurred at the trailing edge of the stator blade, rather than within the large toroidal vortex. This finding is however consistent with clinically reported observations^18^. It was also surprising to observe that the thrombus propagates *upstream*, a phenomenon also observed in our previous simulation of thrombus growth at a site of injury in a blood vessel^9^ and corroborated by experimental observations^30^. This emphasizes the fact that thrombosis is caused by multiple interacting factors as espoused by Virchow’s triad.

An alternative approach to quantify the *thrombogenic potential* of the HeartMate II was reported by Girdhar *et al*.^31^ who employed the thrombogenicity emulation (DTE) methodology based on the shear-loading history of platelets^31^,^32^. This is a Lagrangian approach which computes the cumulative platelet activation level for a large representative population of platelets that traverse through the device. This model thereby combines aspects of both the hemodynamics (flow patterns) and the kinetics of platelet activation. However, it does not include a mechanism for deposition, and therefore does not predict the location within the pump where platelets are likely to deposit or accumulate. This model is also unable to distinguish between “bad” and “good” shear stress. On the one hand, shear exposure has an agonistic effect on platelet activation, but on the other hand it is well known that shear stress is needed to remove or clear agonists or platelets from regions of stagnation. This is often referred to as “washing” of blood-contacting surfaces^33^,^34^,^35^,^36^. The model presented in this report, in contrast, was able to predict that the impeller is free of thrombus while the upstream region experiences unbounded thrombus deposition – despite a similar concentration of active platelets in both regions.

From a design perspective it is *impossible* to create a rotodynamic pump with a perfectly laminar, attached flow field devoid of supra-physiological shear stress, while still performing its useful and intended function of generating pressure and flow. Therefore, every design must seek a compromise. For example, one unsolved challenge is to assess the influence of flow pulsatility caused by the cyclical contribution of the heart. For portions of the cardiac cycle the flow may be well behaved and attached, while during portions of the diastolic phase, the flow may become chaotic^21^. A confounding question for the designer is to determine whether the degree of chaotic flow is tolerable and for what period of time. The numerical simulation here provides a tool to address this challenge. For example, the time-course of thrombus growth in the upstream region, illustrated in Figs 5 and 6, indicates that once there is a critical amount of deposition, there is a positive-feedback effect that causes the platelets to deposit at a greater rate. This is due to the increased concentration of adhesive sites for incoming platelets and the release of platelet synthesized agonists, including ADP and TxA2. Likewise, the absence of thrombus deposition in the impeller can be explained by examining the relative influence of the transport terms of the equations which suggest that there is an equilibrium between deposition and erosion (washing) that interrupts this positive feedback. Therefore, this model could be used to simulate various scenarios of flow pulsatility to determine the critical conditions in which the rate of washing exceeds the rate of accumulation or deposition.

The model used in this study was the product of many years of evolution and has grown rather complex, preventing us from presenting all the details here. The interested reader is referred to a recent validation study by Wu *et al*. which explains all of the mathematical terms, origins of the coefficients, and the main assumptions^9^,^10^. Despite its sophistication, we acknowledge several remaining limitations, including (1) omission of plasma skimming, (2) high computational cost, (3) lack of experimental/clinical validation on the effect of heparin and surface chemistry on pump thrombosis and (4) absence of some potentially relevant pathways of coagulation as well as thrombolysis. The first assumption is justified throughout the majority of the flow field; however, cellular trafficking in regions of small features, such as crevices at the interface between rotating and non-rotating bearing elements, may possibly enhance the concentration of platelets near the surface^9^. Due to the fact that crevices may serve as a nidus for thrombus growth, the study of their effect will receive attention in our future work. The high computational cost of the model limited the number of simulations performed in this study, as well as their duration. Therefore, future work will also explore the possibility of substituting reduced-order models for some of the mechanisms to speed up the numerical process. This will also permit simulations to be performed for longer durations and possibly achieve an equilibrium wherein thrombus growth subsides. We will also consider a design-of-experiments approach to explore the matrix of independent variables – related to flow pulsatility, surface chemistry, hematology, and anticoagulation – more systematically and efficiently. Such a parametric study will provide additional validation of the model for a wider range of conditions beyond those presented here. We recognize one additional limitation of this study is our choice to scale the reaction rate for the sake of computational convenience (i.e. reduce the simulation to 5 days). However, the effect is limited due to the magnitude of the Damkohler (Da) number which exceeds 10^6^ for all 10 species.

We also acknowledge that this study focused on one specific device, the HeartMate II, which will inevitably be supplanted by next-generation devices with a lower incidence of thrombosis. However, we believe that the lessons learned from this study are readily applicable to many other rotodynamic VADs, for example, the HVAD (HeartWare, Framingham, MA)^37^,^38^ which reported in April of 2015 a stroke rate of 27% per patient-year and an astonishing 45% stroke rate at 2 years^38^. Most recently, complications of thrombosis caused HeartWare to interrupt the clinical trial of their next-generation MVAD device^8^,^39^. For those VADs currently in clinical use, the model may be used by physicians to explore the influence of hypothetical interventions, such as a change in speed, introduction of pulsatility, hemodilution, and various anticoagulation regimens. We also hope that device developers and manufacturers would find this thrombosis model useful for optimizing future ventricular assist devices that are increasingly safe and effective.

## Additional Information

**How to cite this article**: Wu, W.-T. *et al*. High fidelity computational simulation of thrombus formation in Thoratec HeartMate II continuous flow ventricular assist device. *Sci. Rep.* **6**, 38025; doi: 10.1038/srep38025 (2016).

**Publisher's note:** Springer Nature remains neutral with regard to jurisdictional claims in published maps and institutional affiliations.

## Figures and Tables

**Table 1:** Boundary conditions for platelets and biochemical species.

Species	Units	Inlet Condition	Boundary Conditions(Flux) at Pump Surfaces
[RP]	PLTm^−3^	3.0 × 10^14^	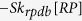
[AP]	PLTm^−3^	0.01[RP]	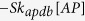
[a_pr_]	nmol^−3^	0.0	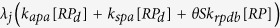
[a_ps_]	nmol^−3^	0.0	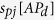
[PT]	nmol^−3^	1.1 × 10^−6^	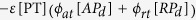
[T]	Um^−3^	0.0	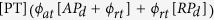
[AT]	nmol^−3^	2.844 × 10^−6^	0.0
[RP_d_]	PLTm^−3^	0.0	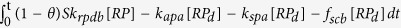
[AP_d_]	PLTm^−3^	0.0	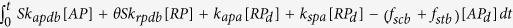
[AP_s_]	PLTm^−3^	0.0	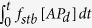

See Wu *et al*.^9^ for explanation of the values and physical meanings of these terms.

**Table 2:** Thickness of thrombus (mm) after 6.25 hours at different axial positions (See Fig. 9).

	FLOW	A	B	C	D	E	F
Hub	2.0 LPM	0.155	0.170	0.190	0.200	0.05	<0.001
	4.51 LPM	0.105	0.150	0.152	0.166	0.019	0.000
	6.0 LPM	0.065	0.100	0.123	0.120	<0.001	0.000
Shroud	2.0 LPM	0.200	0.050	0.360	0.370	0.000	<0.001
	4.5 LPM	0.024	0.060	0.200	0.200	0.000	0.000
	6.0 LPM	0.000	0.060	0.120	0.190	0.000	0.000
Blade	2.0 LPM	N/A	0.140	N/A	N/A	N/A	0.050
	4.5 LPM	N/A	0.090	N/A	N/A	N/A	0.000
	6.0 LPM	N/A	0.090	N/A	N/A	N/A	0.000
